# A case of post COVID‐19 subacute thyroiditis

**DOI:** 10.1002/ccr3.6092

**Published:** 2022-07-19

**Authors:** Mohamed Sadoon Al‐Shammaa, Ahmed Saad Abdlkadir

**Affiliations:** ^1^ Department of Nuclear Medicine Radiotherapy and Nuclear Medicine Hospital Baghdad Iraq; ^2^ Department of Nuclear Medicine King Hussein Cancer Center Amman Jordan

**Keywords:** case report, post COVID‐19 thyroiditis, subacute thyroiditis, thyroid scintigraphy

## Abstract

Subacute thyroiditis is a type of thyroid disease that occurs after a viral infection. This is usually a temporary condition associated with neck pain and may be accompanied by sore throat and flu‐like symptoms. We present a case of post COVID‐19 subacute thyroiditis and review its presentation and outcome.

## Graphical abstract

The clinical presentation and potential complications of post COVID‐19 thyroiditis are still being elucidated in the literature. Treatment for this condition should be individualized based on the disease severity and include a variety of options.

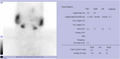

## 1 INTRODUCTION

Subacute thyroiditis is a self‐limiting inflammatory condition of the thyroid gland. The condition is more common in women, and it typically manifests with neck pain and thyrotoxicosis.^1^ The clinical features of the disease typically include symptoms of malaise, neck pain, and fatigue after a viral infection. It is noteworthy that cases of recurrent subacute thyroiditis have been reported previously in the literature.^2^ Thyroid follicles are frequently invaded by viruses, leading to destruction of the basement membrane and rupture of follicular cells. During the first outbreak of COVID‐19 virus, subacute thyroiditis had been reported as a complication that may occur in patients without any previous thyroid disorder.^3^

## 2 CASE PRESENTATION

On November 2020, a 53‐year‐old female patient came to the Nuclear Medicine Department complaining of palpitations, sweating, and agitation. She also reported neck pain for the past 3 days. Her past medical history was unremarkable, except that she had a resolved COVID‐19 infection 10 days before the onset of her new symptoms. Her previous COVID‐19 infection was diagnosed by PCR with overall mild disease pattern that did not require hospitalization. During that time, the patient only reported having symptoms of sore throat and low‐grade fever that continues for about 9 days. The patient also denied any previous personal or family history of autoimmune or thyroid diseases.

**FIGURE 1:**
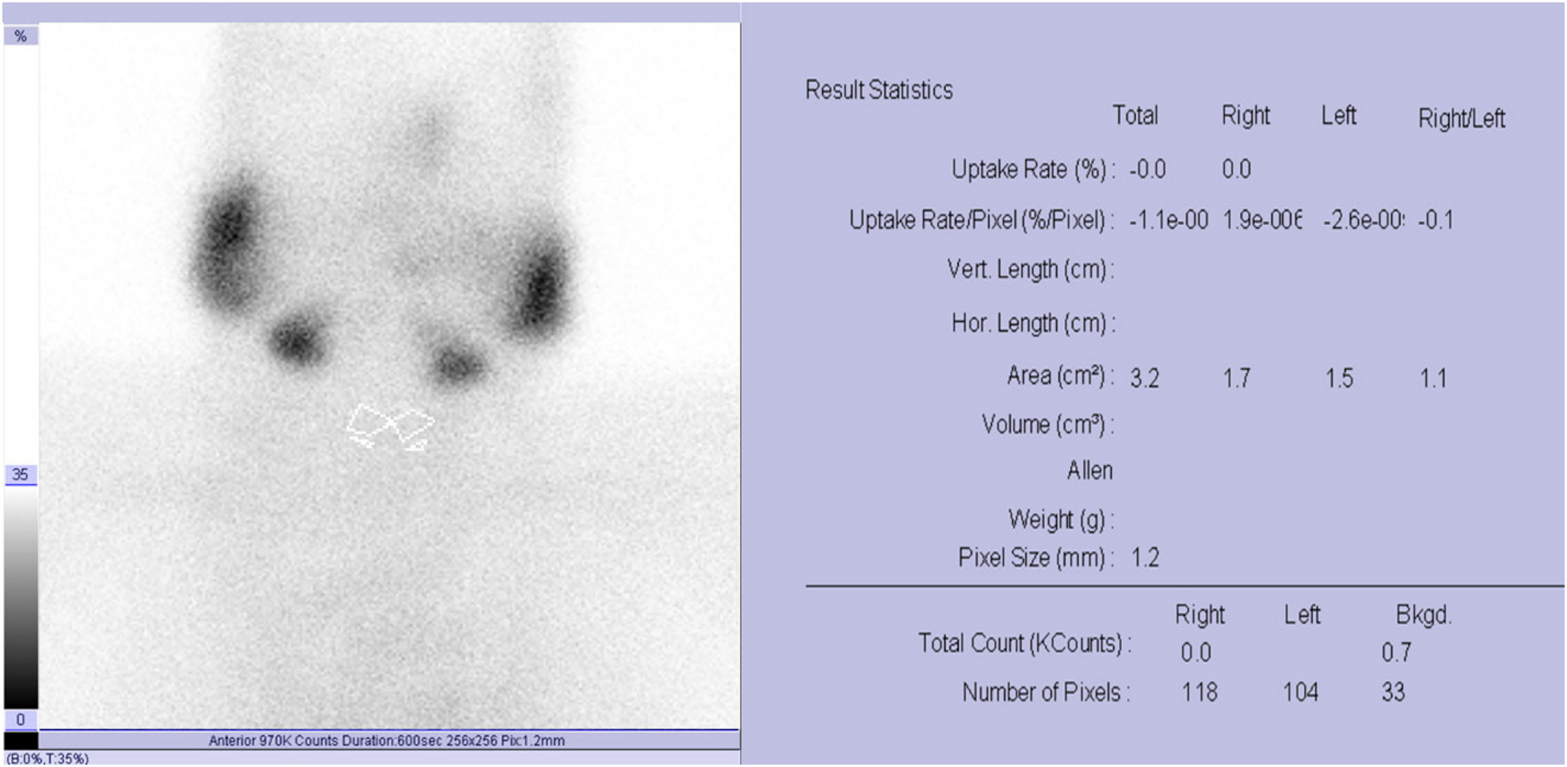
^99m^Tc‐thyroid scan and uptake showed no uptake at thyroid gland indicative of subacute thyroiditis

On examination, she had a sore and soft neck, an increased pulse (134 bpm), and tremors. She had previously conducted lab tests to track her condition after COVID‐19 infection. All of these test results are reflecting the clinical picture of COVID‐19. Thyroid function test (TFT) was requested to rule out thyrotoxicosis (Table 1). Thyroid antibodies were also ordered and performed in an outside facility (Table 2). Neck ultrasound was also requested but the patient was reluctant to perform this imaging modality. The results of the TFT test along with thyroid antibodies values showed that this patient might have a new onset of thyroiditis. In order to reach a definitive diagnosis, a ^99m^Tc thyroid scan and uptake was performed, and it was found that the patient's thyroid gland had below‐normal uptake values (Figure 1). This was indicative of subacute thyroiditis.

**TABLE 1:** Thyroid function test

Thyroid function test	Value	Normal range
T3	3.69 nmol/L	1.20–3.10 nmol/L
T4	234.2 nmol/L	60–181 nmol/L
Free T3	13.8 pmol/L	2.76–11.5 pmol/L
Free T4	31.6 pmol/L	10.3–22.7 pmol/L
TSH	0.021 mIU/L	0.27–4.20 mIU/L

**TABLE 2:** Thyroid antibodies level

Thyroid antibodies	Value	Normal range
Anti‐thyroperoxidase Ab	13.7 U/ml	<9 U/ml
Anti‐thyroglobulin Ab	26.8 U/ml	<18 U/ml
TSH receptor Ab	1.24 IU/L	<1.75 IU/L

The patient was kept on routine monthly follow‐up and was maintained on beta‐blockers with a low dose of analgesia. After 3 months of therapy, the patient clinical condition improved and thyroid function returned back to normal values.

## 3 DISCUSSION

Subacute thyroiditis usually occurs as a post‐viral inflammatory reaction, leading to the destruction of thyroid follicular cells due to infiltration of lymphocytes. Mumps, coxsackie, adenovirus, and influenzae are most commonly associated viruses with causing subacute thyroiditis.^4^

Several case reports and case series have recently been published linking subacute thyroiditis to a viral complication of COVID‐19.^3^, ^5^, ^6^, ^7^

Chen et al. performed a retrospective study analyzing TFT in 50 patients with confirmed COVID‐19 infection and reported that 28 patients (56% of them) had a TSH below the normal result and that the degree of TSH decrease was dependent on the severity of the COVID‐19 infection.^5^

TSH and free T4 levels may be found normal but below baseline values at time of COVID‐19 infection in euthyroid patients. This was noted and concluded in a cohort study conducted by Khoo et al. during the first outbreak.^6^ Gradual recovery towards baseline values were noted during the follow‐up period.^6^

Post‐COVID‐19 thyroiditis is usually characterized by neck pain, palpitation, tremor, fever, and fatigue.^3^, ^5^, ^6^, ^7^ Brancatella et al. reported a case of subacute thyroiditis after SARS‐CoV‐2 infection.^7^ The patient's signs and symptoms included a slightly increased heart rate and a painful and enlarged thyroid gland on palpation.^7^ At laboratory examinations, free thyroxine and free triiodothyronine levels were high, TSH was undetectable, and inflammatory markers and white blood cell count were elevated. There were hypoechoic areas on neck ultrasound detected in both bilateral and diffuse forms. The case was diagnosed as subacute thyroiditis, and the patient was started on prednisone.^7^ The neck pain and fever resolved within 2 days, and the remaining symptoms also resolved within 1 week.^7^ In just 40 days, thyroid function normalized and inflammation markers were trending down.^7^

Mattar et al. reported a case of a hospitalized patient who developed subacute thyroiditis in association with SARS‐COV‐2 infection.^3^ The patient had tachycardia and anterior neck pain. Thyroid function tests revealed that the patient had hyperthyroidism.^3^ This is consistent with ultrasonographic evidence of subacute thyroiditis. Treatment with 10 weeks of corticosteroid therapy resulted in a complete resolution of signs and symptoms of subacute thyroiditis.^3^

A combination of corticosteroids, beta‐blockers, and analgesics is the most commonly used treatment for patients with post‐COVID‐19 thyroiditis.^7^ In one reported case, the combination of hydroxy‐chloroquine and prednisone was used.^8^ In some cases, there was no need to give the patient corticosteroid treatment.^9^ In our case, the patient's symptoms were mild and improved using only beta‐blockers, with a low dose of analgesia.

## 4 CONCLUSION

Subacute thyroiditis should be considered as a possibility in COVID‐19 patients and if present must be treated by combination of short course corticosteroids, beta‐blockers (mostly propranolol), and analgesia to control the patient's symptoms and achieve full recovery. Still future studies and reports will help reach more definitive guidelines to control this complication and achieve the best outcome.

## AUTHOR CONTRIBUTIONS

MS was the principal investigator of this manuscript and approved the final manuscript. MS and ASA were responsible for the study concept, design, writing, reviewing, and editing of the manuscript in its final form.

## CONFLICT OF INTEREST

The authors declare that they have no conflict of interest.

## ETHICAL APPROVAL

We conducted the research following the Declaration of Helsinki. Ethical permission was granted by [Department of Nuclear Medicine, Medical City, Baghdad 10,049, IRAQ]. The participant consent form was secured.

## CONSENT

Written informed consent was obtained from the patient for publication of the case report and accompanying images.

## Data Availability

The data that support the findings of this study are not publicly available due to privacy restrictions. The data are available upon reasonable request from the corresponding author.
